# Complete cardiac regeneration in a mouse model of myocardial infarction

**DOI:** 10.18632/aging.100526

**Published:** 2012-12-31

**Authors:** Bernhard Johannes Haubner, Martyna Adamowicz-Brice, Sanjay Khadayate, Viktoria Tiefenthaler, Bernhard Metzler, Tim Aitman, Josef M. Penninger

**Affiliations:** ^1^ IMBA, Institute of Molecular Biotechnology of the Austrian Academy of Sciences, 1030 Vienna, Austria; ^2^ MRC Clinical Sciences Centre and Imperial College, London W12 0NN, UK; ^3^ Department of Internal Medicine III (Cardiology), Innsbruck Medical University, 6020 Innsbruck, Austria

**Keywords:** Heart regeneration, cell cycle, heart disease

## Abstract

Cardiac remodeling and subsequent heart failure remain critical issues after myocardial infarction despite improved treatment and reperfusion strategies. Recently, complete cardiac regeneration has been demonstrated in fish and newborn mice following resection of the cardiac apex. However, it remained entirely unclear whether the mammalian heart can also completely regenerate following a complex cardiac ischemic injury. We established a protocol to induce a severe heart attack in one-day-old mice using left anterior descending artery (LAD) ligation. LAD ligation triggered substantial cardiac injury in the left ventricle defined by Caspase 3 activation and massive cell death. Ischemia-induced cardiomyocyte death was also visible on day 4 after LAD ligation. Remarkably, 7 days after the initial ischemic insult, we observed complete cardiac regeneration without any signs of tissue damage or scarring. This tissue regeneration translated into long-term normal heart functions as assessed by echocardiography. In contrast, LAD ligations in 7-day-old mice resulted in extensive scarring comparable to adult mice, indicating that the regenerative capacity for complete cardiac healing after heart attacks can be traced to the first week after birth. RNAseq analyses of hearts on day 1, day 3, and day 10 and comparing LAD-ligated and sham-operated mice surprisingly revealed a transcriptional programme of major changes in genes mediating mitosis and cell division between days 1, 3 and 10 postnatally and a very limited set of genes, including genes regulating cell cycle and extracellular matrix synthesis, being differentially regulated in the regenerating hearts. We present for the first time a mammalian model of complete cardiac regeneration following a severe ischemic cardiac injury. This novel model system provides the unique opportunity to uncover molecular and cellular pathways that can induce cardiac regeneration after ischemic injury, findings that one day could be translated to human heart attack patients.

## INTRODUCTION

Cardiovascular diseases are the most common cause of death in North America and Europe [1] killing more than 860,000 people annually in the United States [2, 3]. Moreover, 80 million people in the USA are estimated to suffer from cardiovascular diseases [2, 3]. Known or associated causes of cardiovascular disease include diabetes mellitus smoking, high cholesterol, hyper-tension, inflammation, overweight and obesity, or physical inactivity [2, 3]. Although there have been great advances in the understanding of heart failure in recent decades [4] and acute treatment of heart attacks has markedly improved survival, one essential key issue is how to potentially regenerate the heart after injury.

It had been assumed that cardiac regeneration is impossible; however, cardiac progenitor cells have been found at low frequency even in the mammalian heart and major attempts have been made to either use the potential of progenitor cells for cardiac repair [5, 6] or to transdifferentiate cardiac stroma cells into cardiomyocytes [7, 8]. Pioneering experiments demonstrated that fish can completely regenerate the heart following resection of the heart apex [9], a finding that has spurned multiple studies using fish as a model organism [10-14]. Intriguingly, it has also been recently shown that newborn mice, but not mice beyond one week of age, can regenerate the cardiac apex following resection [15]. However, it remained entirely unclear whether mammals can regenerate the heart following a complex cardiac ischemic injury that affects central cardiac regions of the heart, is associated with massive cell death and tissue damage.

Here we report the development of a cardiac ischemia injury model in newborn mice. Intriguingly, despite massive tissue damage in this model, we observed complete morphologic and functional cardiac repair, indicating that heart regeneration is possible following complex myocardial infarction injury in mammals.

## RESULTS

### A novel model system for irreversible cardiac injury in newborn mice

**Figure 1:**
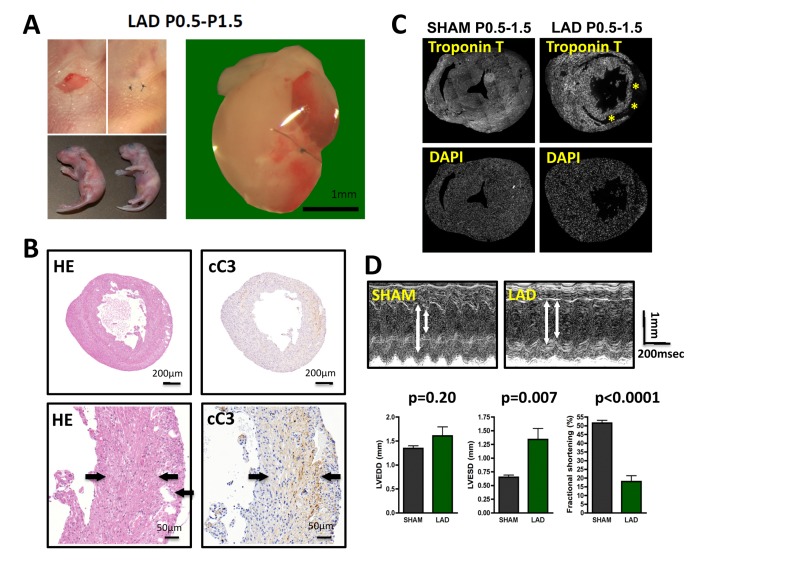
LAD ligation in newborn mice results in massive cardiac damage **(A)** Incisions into the left chest wall were used to open the chest cavity and irreversibly ligate the left anterior descending artery (LAD) in newborn mice. Representative images are shown. In Sham-operated mice the chest cavity was opened, without LAD ligation. **(B-D)** Massive cardiac damage 24 hours after the initial LAD ligation as demonstrated by H&E staining **(B)**, immune-detection of cleaved Caspase 3 (cC3) **(B)**, loss of TroponinT expression (negative = infarction area, asterisk) **(B)**, and echocardiography **(D)**. Arrows in B indicate infarction area. Data from sham operated newborn mice are shown as controls. In **(B)** and **(B)** images are representative of 5 mice analyzed. Data in **(D)** are shown as mean values +/− SEM (n=6 per group). Arrows in **(D)** indicate LVESD and LVEDD. Probability values were determined using a Mann-Whitney U Test.

To examine whether there is cardiac regeneration in newborn mammalian heart following a complex ischemic injury, i.e. myocardial infarction, we established a protocol for left anterior descending artery (LAD) ligation in one-day-old mice, i.e. postpartum P0.5 to postpartum P1.5 (Fig. 1A). Using hypothermia anesthesia [16], the LAD artery was irreversibly ligated and subsequent tissue remodeling and regeneration were assessed using serial histological sectioning of the hearts combined with immunohistochemistry and lineage tracing studies.

LAD ligation on P0.5 resulted in massive tissue damage determined 24 hours after the initial injury as assessed by histological damage, Caspase 3 activation (cleaved caspase 3) to assess cell death (Fig. 1B), and Troponin T staining as a measure of cardiomyocyte damage (Fig. 1C). Moreover, echocardiography at 24 hours after injury revealed markedly altered left ventricular end-diastolic diameters (LVEDD) and left ventricular end-systolic diameters (LVESD) resulting in massive impairment in fractional shorting (Fig. 1D), a measure for cardiac function [17]. Thus, LAD ligation in newborn mice results in massive cardiac tissue damage and consequent impaired heart functions.

### Complete cardiac regeneration following massive cardiac infarction in newborn mice

**Figure 2:**
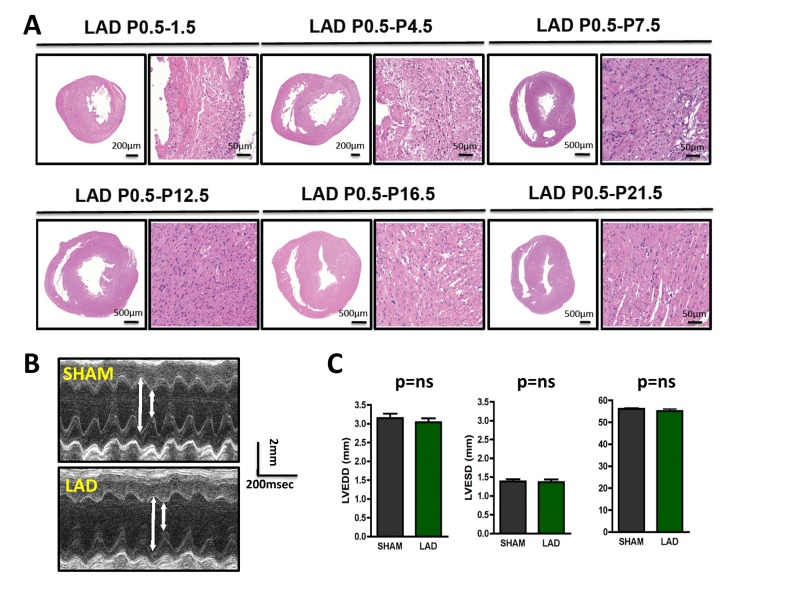
Time-course of cardiac regeneration **(A)** Time-course of regeneration. Whole hearts and magnifications at the infarction areas are shown on day P1.5, P4.5, P7.5, P12.5, P16.5, and P21.5 following LAD ligation of newborn mouse hearts at P0.5. Images are representative of 4 mice analyzed. Note the complete regeneration of the infarction zone starting ~ day P7.5. **(B-C)** Cardiac echocardiography of 3 months old mice that where either sham-operated on P0.5 or received LAD ligation on P0.5. Representative M-mode echocardiograms are shown in **(B)**. Arrows show LVESD and LVEDD. Data in (**C)** show mean values +/− SEM of LVEDD, LVESD, and Fractional shortening (n = 4 per group). ns, not significant (Mann-Whitney U Test).

We next followed the time course of cardiac tissue damage. Four days following initial LAD ligation in newborn mice, we still observed marked tissue injury. Remarkably, starting around day 7 we observed nearly complete cardiac regeneration. On the consecutive days of analysis (P12.5, P16.5, and P21.5) we also did not observe any histological sign of residual tissue damage (Fig. 2A). Moreover, echocardiography of 3 months old mice that where ligated on P0.5 showed complete and long-term restoration of cardiac functions as compared to mice that underwent sham surgery (Fig. 2B,C).

**Figure 3:**
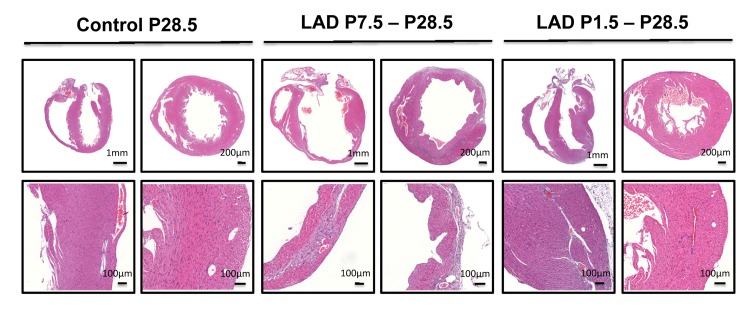
A time window for cardiac regeneration Whole hearts and magnifications at the infarction areas are shown on day P28.5 following LAD ligation of newborn mice at P1.5 and LAD ligation in mice at P7.5 after birth. Images are representative of 6 mice analyzed. Note the complete regeneration of the infarction zone when mice received LAD-ligation on P1.5, whereas no regeneration was seen when mice were LAD-ligated on day P7.5.

To determine the time window for cardiac regeneration following LAD ligation, we performed cardiac infarction experiments in mice at P1.5 and at P7.5 after birth; heart regeneration was determined on day P28.5. Whereas complete regeneration was observed when the left anterior descending artery was ligated in newborn mice, the ischemic damage could not be repaired when LAD ligation was performed in 7.5 day-old mice (Fig. 3). Thus, newborn mice have the capacity for complete morphological as well as functional cardiac regeneration following massive ischemic heart damage and this capacity is lost by day 7.5 of age.

### Cell fate mapping of regenerating cardiomyocytes

**Figure 4:**
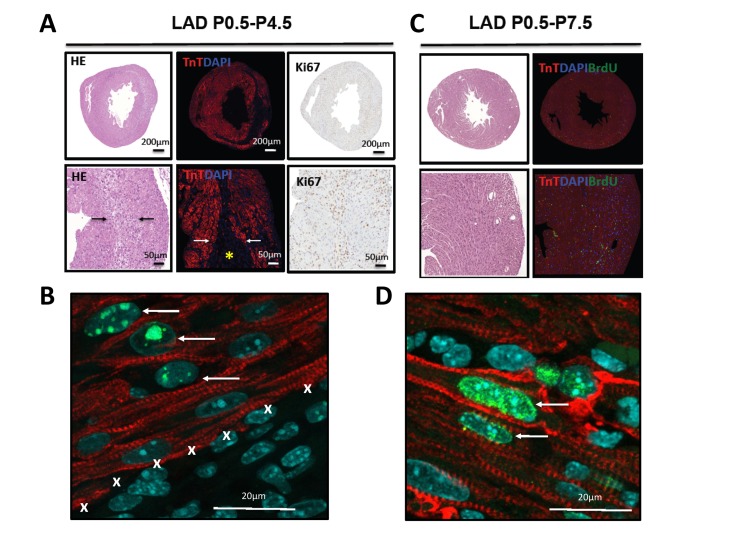
Evidence for proliferation in cardiomyocytes **(A)** Whole hearts and magnifications are shown for hearts LAD-ligated on P0.5 and harvested on P4.5. Sections were stained with H&E, immunoflourescently labeled for TroponinT and DAPI, and for the proliferation marker Ki67. Images are representative of 4 mice analyzed. Note the border zone of the infarction area (arrows) and the complete loss of TroponinT positive cardiomyocytes in the infarction zone (asterisk). **(B)** High magnification (63 fold) of a heart stained for TroponinT, phospho-Histone3 (Ser10, pH3), and DAPI after 4 days of LAD ligation (P0.5-P4.5). Note the double pH3 and TroponinT positive cardiomyocytes (arrows) at the border zone of the area of infarction (marked by X). **(C)** Whole hearts and magnifications are shown for hearts LAD-ligated on P0.5 and harvested on P7.5. Sections were stained using H&E and immunoflourescently labeled for TroponinT, DAPI, and BrdU as a marker for DNA replication. No residual TroponinT negative area is visible, indicating complete cardiac regeneration. Images are representative of 4 mice analyzed. **(D)** High magnification of a heart stained for TroponinT, BrdU, and DAPI after 7 days of LAD ligation (P0.5-P7.5). Arrows point at BrdU incoperation in TroponinT positive cardiomyocytes.

Staining for TroponinT to image loss of cardiomyocytes and for the proliferation marker Ki67 showed marked proliferation of cells in the infarction zone on P4.5 of mice that were LAD-ligated shortly after birth (P0.5) (Fig. 4A). This finding was confirmed using phospho-H3 immunostaining. Importantly, we observed phospho-H3 staining in TroponinT positive cardiomyocytes at this time point indicating that cardiomyocytes at the edge of the infarction zone had entered the cell cycle (Fig. 4B). BrdU labeling further confirmed that at P7.5 the TroponinT^+^ cardiomyocytes that were present in the regenerated initial infarction zone had undergone DNA replication (Fig. 4C,D). Thus, the infarction zone is repopulated by cardiomyocytes that exhibit molecular marks of cell cycle progression.

**Figure 5:**
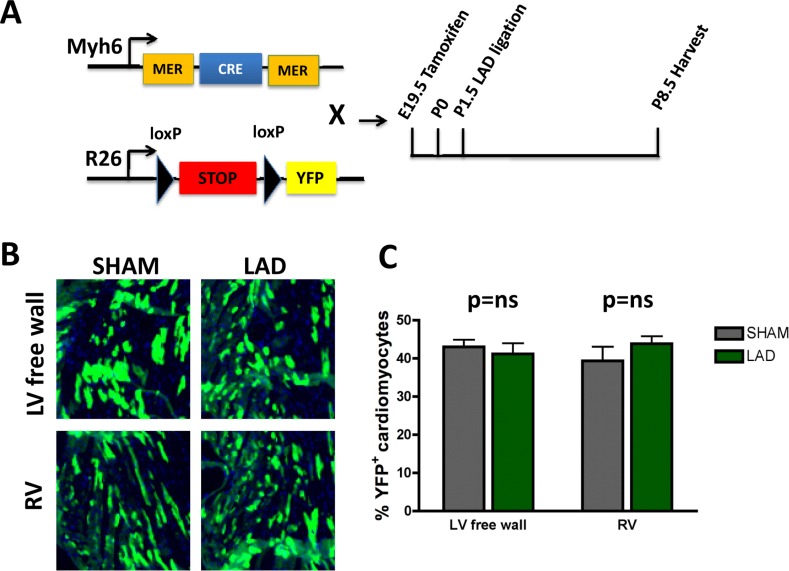
Fate mapping of cardiomyocytes that repair heart attacks **(A)** Schematic representation of the lineage tracing experiment. Homozygous female *Rosa26-loxPSTOPloxP-YFP* reporter mice were crossed with male *Myh6-MerCreMer* mice. Tamoxifen (20mg/kg in peanut oil) was administered intraperitoneally into pregnant females at E19.5. Newborns were transferred to foster mothers immediately after birth. LAD ligation was performed on P1.5 and hearts were harvested 7 days later on P8.5. **(B-C)** No significant difference of YFP positive cardiomyocytes in the area at risk (left ventricular free wall = LV free wall) or in the remote myocardium, i.e. right ventricle (RV), between LAD-ligated and SHAM operated mice (n=4 per group). ns, not significant (Mann-Whitney U Test).

To trace the lineage of the regenerated cardiomyocytes that have repaired the ischemic injury area, i.e. already differentiated cardiomyocytes that can enter the cell cycle and thus repair the injury vs recruitment of progenitor cells [6], we performed lineages tracing experiments using two mouse lines (Fig. 5A). This genetic set-up allowed us to induce, via Tamoxifen, YFP expression in cardiomyocytes and thereby follow their fate in the injury zone, a model system previously used to mark cardiomyocyte fate [15, 18]. In order to extend the time-window between the tamoxifen administration and LAD ligation we injected tamoxifen into pregnant mothers at E19.5. This strategy led to a reduced but constant labeling efficiency of about 40%. In the neonates we then ligated the LAD and harvested the hearts 7 days later on P8.5 (Fig. 5A). We could never detect any significant difference in the relative number of YFP positive cardiomyocytes between sham and LAD operated mice especially in the area of the left ventricular free wall (Fig. 5B,C). Our data confirm and reiterate earlier data shown in neonatal mice [15] and recently adult mice [19]; following cardiac injury, cardiomyocytes appear to re-enter the cell cycle, proliferate, and thereby replace and repair the damaged cardiac tissue area.

### Changes in cell cycle genes in the postnatal and regenerated heart

**Figure 6:**
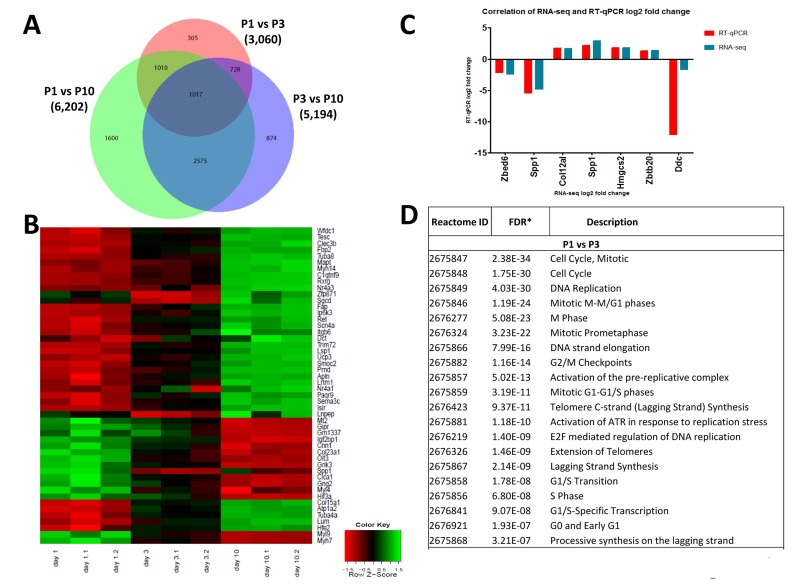
Differentially expressed genes in the left ventricle of postpartum P1, P3, and P10 mice **(A)** Venn diagram of differentially expressed genes in the left ventricle of non-manipulated P1, P3 and P10 mice. Numbers of genes differentially expressed comparing P1 vs P3, P1 vs P10, and P3 vs P10 are indicated. **(B)** Heat map showing the progression of the 50 most differentially expressed genes in individual mice between P1, P3 and P10. Most transcripts progress in a linear, or approximately linear, fashion from P1 to P3 and P10 are shown. However, a small set of genes robustly shows an expression increase from P1 to P3 and then a decrease from P3 to P10, whereas another small gene set shows a decrease and then an increase. **(C)** Validation of RNA-seq results using qRT-PCR. Data indicate a strong correlation between RNAseq data and qPCR expression analyses for the majority of the validated genes accept *Ddc*, which could be caused by a poor reproducibility of this gene assay. **(D)** Reactome analysis of differentially expressed genes between P1, P3 and P10 mice showing that the most differentially expressed gene between these stages of the development belong to mitosis and cell cycle categories.

To assess possible changes in gene expression profiles in the heart we first performed RNA-seq at P1, P3, and P10 of non-manipulated mice to provide a list of comprehensive transcriptional changes of the early postnatal heart. Remarkably, we observed changes in gene expression of thousands of genes when comparing hearts from P1, P3 and P10 after birth (Fig. 6A, Suppl. Fig. 1, Supplementary Table 1A-C). We next constructed a heat map to determine progression of the top 50 differentially expressed genes, in individual mice, between P1, P3 and P10 (Fig. 6B). Expression of most genes progressed in a near linear fashion from P1 to P3 and P10 (expression either going up or down during this time course), whereas a small number of genes robustly showed an increase from P1 to P3 and then a decrease from P3 to P10 (or vice versa). The most upregulated genes between P1 and P3, all more than 10 fold, are: *Hrg, Uox, Fabp1, Serpina1C, Pcp4, Afp, Tnmd, Sprr2a1, Gc, Alb, Serpina1a*, and *Apob*.

Among the downregulated genes, *NPPA* (atrial natriuretic factor) is downregulated 9-fold in P1 vs P3 hearts. Comparing P3 and P10 hearts we found for instance marked upregulation of *Hemicentin 2*, involved in the organization of hemidesmosomes [20], or *NR4A3*, a nuclear steroid hormone receptor [21], whereas *HIF3A* and the stem cell/progenitor marker *Nanog* were ~ 10-fold downregulated in P10 hearts. Differential expression of 6 candidate genes was confirmed by qRT-PCR (Fig. 6C). Most importantly, reactome analyses revealed that the genes changing postnatally are almost all mitosis or cytokinesis genes (Fig. 6D, Supplementary Table 2). Thus, one key transcriptome signature of the postnatal heart is a massive change in the mitotic machinery, correlating very closely with shutting down of cell cycle.

**Figure 7:**
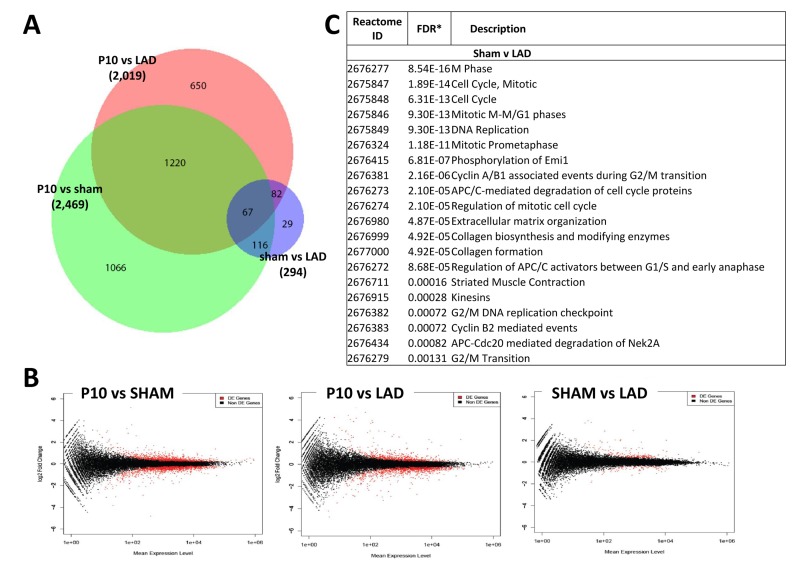
Differentially expressed genes between sham-operated and LAD-ligated mice left ventricles **(A)** Venn diagram of differentially expressed genes in the left ventricle of non-manipulated, LAD-ligated and sham-operated mice, all from P10. The numbers of genes differentially expressed comparing non-manipulated P10 vs LAD-ligated hearts, non-manipulated P10 vs sham-operated heart, and LAD-ligated vs sham-operated hearts are indicated. **(B)** MA plots showing the distribution of gene expression plotted against log(fold change) for each gene in each pair-wise comparison of P10, LAD-ligated and sham-operated mice. Red dots indicate differentially expressed genes (Padj < 0.05), black dots indicate non-differentially expressed genes. **(C)** Reactome analysis of differentially expressed genes between sham-operated and LAD-ligated left ventricles at P10 revealing that the majority of these genes are annotated to the regulation of and mitosis cell cycle control as well as extracellular matrix and collagen pathways.

We next compared the cardiac transcriptome of non-manipulated, LAD-ligated, and sham-operated mice in 10 day-old mice (P10). Of note LAD ligation or sham-operations were performed at P1, i.e., we analyzed a time-point where we already observed complete regeneration of the damaged cardiac tissue. Intriguingly, whereas there were large differences in the transcriptomes as compared to non-manipulated mice, remarkably we detected only a very small number of differentially expressed genes (294 genes) at P10 comparing LAD-ligated and sham-operated mice (Fig. 7A,B; Supplementary Table 1D-F). The genes names, fold changes, and p values for all of the 294 differentially expressed genes are listed in Supplementary Table 3. Comparison of the 294 transcripts that showed statistically significant differential expression between LAD and sham-operated mice showed that most genes were related to cell cycle or mitosis, but also included genes on the extracellular matrix and collagen synthesis pathways (Fig. 7C and Supplementary Table 2). Thus, as compared to sham-operations, the regenerated LAD-ligated hearts show remarkably few transcriptional changes, but still carry a gene expression signature indicating that the cell cycle machinery has not been entirely switched off yet in the repaired cardiac tissue.

## DISCUSSION

Efficient heart regeneration has been one of the prime visions for cardiology [22, 23]. First attempts to use bone marrow derived-progenitor cells have proven inconsistent [24, 25] and recently new avenues have been taken to identify novel cardiac progenitor populations [26] or to activate, for instance, epicardial progenitors for heart regeneration [27]. Moreover, it has been recently reported that fibroblasts in the damaged myocardium can be reprogrammed into cardiomyocytes [7, 8] and regeneration can be initiated by pre-existing cardiomyocytes [19]. However, in most cases regeneration has proven to be limited in terms of the repaired areas and in terms of restoration of heart functions [7, 8, 28].

The discovery that fish can regenerate the heart following resection of the tip of the heart was a revelation because it introduced the concepts that complete heart regeneration might be possible [9]. That cardiac regeneration following apex resection is also possible in newborn mouse heart [15] further supported this notion. Our data now show that newborn mice have also the capacity to completely repair, within 7 days, a complex injury following a severe cardiac ischemic insult. Lineage tracing suggests that, at least in part, such regeneration is mediated via cell cycle entry of pre-existing cardiomyocytes. Based on our gene expression data it appears that neonatal cardiomyocytes maintain the capacity to proliferate whereas this capacity is lost around 7 days after birth. Whether this loss correlates and is possibly regulated via binucleation of cardiomyocytes, which in mouse occurs within the first week after birth [29], needs to be determined.

Intriguingly, although we showed that expression of thousands of genes comparing the neonatal heart to cardiac tissue on postpartum days 3 and 10, we found only about 290 differentially expressed genes between sham-operated and LAD ligated hearts assessed on P10. As defined by reactome analyses, most of these differentially expressed genes have been annotated to cell cycle progression, mitosis, or DNA replication. Thus, clearly one of the key mechanisms that permit regeneration vs scarring is the differential regulation of cell cycle genes in the newborn heart. The observation of only a small number of differentially expressed genes between LAD-ligated and sham-operated mice is consistent with the near complete functional recovery of cardiac function in LAD-ligated mice. The finding of differentially expressed genes on the pathway for extracellular matrix and collagen synthesis between LAD-ligated and sham-operated mice is likely indicative of residual healing of the myocardium post-infarction. The control of cell cycle progression in cardiomyocytes is of particular interest not only for cardiac regeneration but also for the fact, that - to our knowledge - not a single cardiomyocyte-derived tumor has ever been reported in the literature. We of course cannot exclude differences in the newborn versus day 7 old mice in cardiac stem cells, inflammatory responses to the tissue damage, or changes in the fibrotic response as also playing a part in the alteration in mitotic potential of the myocardium between postpartum day 1 and day 7.

In conclusion, our model system reveals that complete repair of a myocardial infarction is possible in the newborn mouse. Whether this extends to other mammalian species needs to be tested. Importantly, our model might allow to possibly unlock fundamental mechanisms of regeneration that could be used in the future to also repair adult hearts.

## METHODS

### Ligation of the left anterior descending artery (LAD) in neonates

P0.5, P1.0, P1.5 neonatal and one week old (P7.5) C57BL6J mice were used for the LAD ligation model. First, neonates were briefly sedated by putting the newborns into an isoflurane induction chamber followed by an approximately 4 min long cooling period in ice water. Hypothermia induces a reversible cardiac arrest in pre-weanling rodents and thereby allows for controlled thoracic surgery [16]. Next the experimental animals were taped to a cooling bag in the right lateral position to ensure continued hypothermic anesthesia during the surgical procedure. The skin was cut just a few millimeters below the left foreleg. Next, the Pectorals major et minor muscles were dissected and the thorax opened in the 4^th^ intercostal space. Ligation of the left anterior descending artery was performed with a 10-0 ethilon suture (Ethicon). The musculosceletal thorax and the skin were closed with a 8-0 vicryl suture (Ethicon). Finally, mice were put onto a 38^o^ Celsius heated pad. After confirmation of spontaneous breathing the mice received a dose of subcutaneous buprenorphine (0.05mg/kg). Survival rates in our protocol were above 90% of sham-operated and above 90% of LAD-ligated mice. All animal experiments were approved by the Austrian Ethical Board.

### Histology

The hearts were harvested at defined time points, washed in PBS and then fixed in 4% paraformaldehyde (PFA) overnight at room temperature. Next, the PFA was exchanged with 70% Ethanol and the samples embedded in paraffin and cut at 2.5μm. Paraffin sections were rehydrated and then either stained with hematoxylin and eosin (HE) or further processed for immunohistochemical stainings.

### Immunohistochemistry

2.5μm paraffin sections were rehydrated followed by antigen retrieval in boiling citrate buffer for 20 min. Samples were then stained overnight at 4^o^ Celsius with antibodies against phospho-Histone 3 (Ser10, #04-1093, Millipore), TroponinT (#MS-295, Thermo Scientific), BrdU (#ab6326, Abcam), cleaved Caspase 3 (#9661, Cell Signaling), and Ki67 (#PA0118, Novocastra). Goat anti-mouse Alexa 555 and goat anti-rabbit Alexa 488 were used as secondary antibodies at room temperature for 1 hr. Samples were counterstained with DAPI for 15 min and mounted in DAKO. Yellow fluorescent protein (YFP) was detected on cryosections; hearts were first fixed in 4% PFA overnight and then washed in PBS once. Next, the fixed hearts were stored in 30% sucrose/PBS at 4^o^ Celsius overnight and then embedded in OCT. 10μm sections were cut from the cryo-preserved tissue blocks and stained with an antibody against GFP that crossreacts with YFP (Invitrogen).

### Lineage tracing

Homozygous female YFP reporter mice (Rosa26-loxP STOP loxP-YFP) were crossed with male Myh6-MerCreMer mice. Pregnant females were injected Tamoxifen (Sigma, 20mg/kg dissolved in peanut oil) intraperitoneally on day E19.5. Newborn mice were immediately transferred to a foster mother and the LAD ligation procedure was performed on P1.5. After 7 days of permanent LAD ligation, hearts were harvested on P8.5 and stained for YFP as described above.

### 5-bromo-2-deoxyuridine (BrdU) labeling

LAD ligation was performed on P0.5. Subsequently, 0.03 ml of a 20mg/ml BrdU/0.9%NaCl (Sigma) solution was injected intraperitoneally on P1.5 and P3.5.

### Echocardiography

Myocardial contractilitywas determined by transthoracical echocardiography using a Vevo 770 system (Visualsonics, Toronto). Long-axis 2D-targeted M-mode images of the left ventricle were obtained. Fractional shortening (FS) was calculated from digital images by using a standard formula.

### RNA isolation and RNA-seq library preparation

RNA was extracted from 2-5mg of left ventricular tissue from three P1, P3, P10, sham-operated and LAD-ligated animals without pooling, using Trizol (Invitrogen) according to manufacturer's instructions with an additional purification step by on-column DNase treatment using the RNase-free DNase Kit (Qiagen) to ensure elimination of any genomic DNA. The integrity and quantity of RNA was determined using a NanoDrop 1000 spectrophotometer (Thermo Fisher Scientific) and Agilent 2100 Bioanalyzer (Agilent Technologies). 4μg of total RNA was used to generate RNA-seq libraries using TruSeq RNA sample preparation kit (Illumina) according to the manufacturer's instructions. Briefly, RNA was purified and fragmented using poly-T oligo-attached magnetic beads using two rounds of purification followed by the first and second cDNA strand synthesis. Next, cDNA 3' ends were adenylated and adapters ligated followed by 10 cycles of library amplification. Finally, the libraries were size selected using AMPue XP Beads (Becmkan Coulter) purified and their quality was checked using Agilent 2100 Bioanalyzer. Libraries were run on a single lane per sample of the HiSeq 2000 platform (Illumina) to generate 100bp paired-end reads.

### RT-qPCR

Six differentially expressed genes between the pairwise comparisons were selected for validation by RT-qPCR: *Col12a1*, *Ddc*, *Hmgcs2*, *Spp1*,*Zbed6* and *Zbtb20* and for one housekeeping gene, *Actb*. cDNA was synthesized using iScript cDNA synthesis kit (BioRad) using manufacturers instruction. Primers were designed using Primer 3. Standard curves and fluorescent quantitation PCR were performed using 7900HT Fast Real Time PCR Machine (Applied Biosystems) and cDNA quantified according to the manufacturer's software.

### Differential gene expression analysis

Raw RNASeq reads were aligned with Tophat splice junction mapper [30], version 1.4.1 against UCSC Human genome reference sequence assembly (mm10) and transcript annotations (version NCBI 38.68). Gene based read counts were then obtained using HTSeq count module (version 0.5.3p9). Differential expression analysis was performed on the counts data using DESeq Bioconductor package [31]. The analysis was run with the default parameters. Independent filtering was done on the counts data prior to statistical testing to remove 40 % of the genes with lowest counts. The DESeq package uses a negative binomial model to test for differential expression. Raw p values were then adjusted for multiple testing with the Benjamini-Hochberg procedure. Genes with adjusted p value of 0.05 or less were termed as differentially expressed genes. The analysis was performed using R 2.15. Comparisons were performed between non-manipulated P1, P3 and P10 mouse hearts. LAD-ligated mouse hearts were compared against sham-operated mice samples and also against non-manipulated P10 mouse hearts. Of note, from the 294 differentially expressed genes comparing LAD-ligated vs sham-operated hearts we removed Xist from the list in Supplementary Table 3 because of current experiments performed to assess sex differences between the cohorts.

### Clustering analysis

After detecting differentially expressed genes in the heart, genes that changed in common between all three time points (P1, P3, P10 of non-manipulated mice) were then determined using sdef R package [32]. The mean of the p values for the differentially expressed genes in the three comparisons was taken and the top 50 genes with lowest mean p value were clustered and visualized in a heatmap. Ensembl identifiers were mapped to gene symbols prior to produce the heatmap. Custom scripts were written to perform the clustering and visualization of the data. The analysis was performed using R/2.14.2.

### Reactome pathway analysis

Reactome pathway analysis was carried out on the genes found to be differentially expressed in various comparisons to test for enrichment of pathways in the Reactome database. The Bioconductor package GOseq [33] was used for this enrichment analysis. The package corrects for length bias present in RNASeq data. Raw p values were adjusted for multiple testing using the Benjamini-Hochberg procedure. The analysis was performed on R 2.15.

### Statistical analysis of data

All data are expressed as mean ± SEM. Statistical analyses were performed using the Mann-Whitney U Test (SPSS software). Probability values <0.05 were considered significant. P values for gene expression differences were adjusted with the Benjamini-Hochberg false discover rate.

## SUPPLEMENTARY DATA
















