# Supplementary material for: Complete cardiac regeneration in a mouse model of myocardial infarction
Source: Aging (Albany NY). 2012 Dec 31;4(12):966–77. doi: 10.18632/aging.100526 (PMC3615162; doi:10.18632/aging.100526)
Supplement: Supplementary file 1 [file aging-04-966-s001.pdf]

31. Anders S, Huber W. Differential expression analysis for sequence count data. Genome Biol. 2010; 11:R106.
32. Blangiardo M, Cassese A, Richardson S. sdef: an R package to synthesize lists of significant features in related experiments. BMC Bioinformatics. 2010; 11:270.
33. Young MD, Wakefield MJ, Smyth GK, Oshlack A. Gene ontology analysis for RNA-seq: accounting for selection bias. Genome Biol. 2010; 11:R14.

SUPPLEMENTARY DATA

The Supplementary Tables are found in full version of this manuscript.

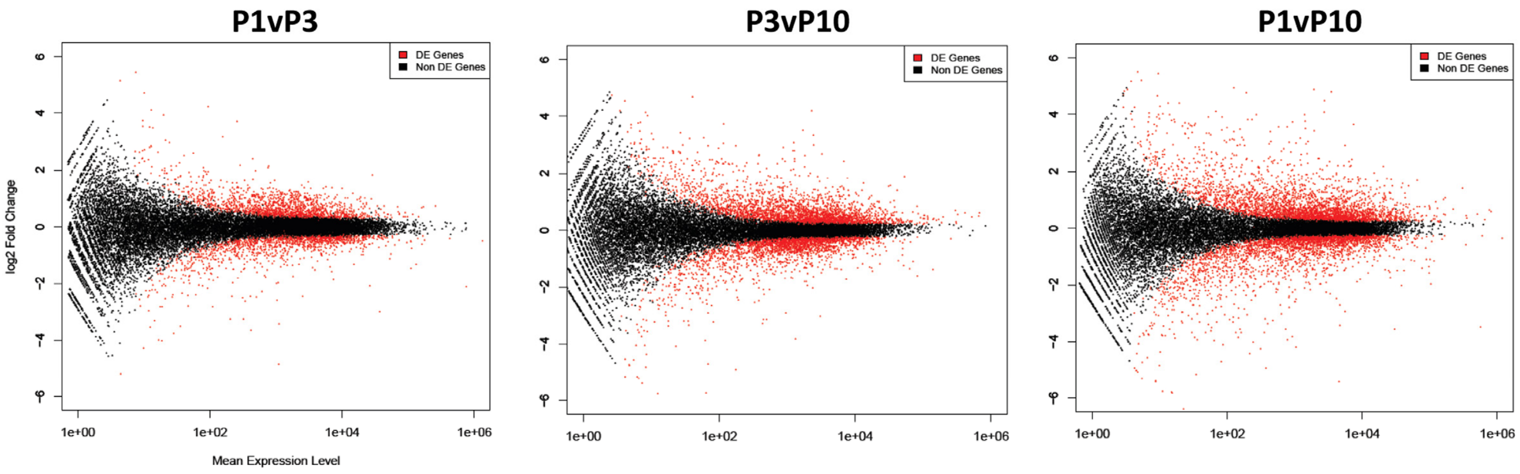

Supplementary Figure 1. MA plots showing the distribution of gene expression plotted against log(fold change) for each gene in each pair wise comparison of P1, P3 and P10. All expression data are from the left ventricle. Red dots indicate differentially expressed genes (Padj < 0.05), black dots indicate non-differentially expressed genes.

.
